# The Role of Circular RNAs in Pancreatic Ductal Adenocarcinoma and Biliary-Tract Cancers

**DOI:** 10.3390/cancers12113250

**Published:** 2020-11-04

**Authors:** Christopher Limb, Daniel S. K. Liu, Morten T. Veno, Eleanor Rees, Jonathan Krell, Izhar N. Bagwan, Elisa Giovannetti, Hardev Pandha, Oliver Strobel, Timothy A. Rockall, Adam E. Frampton

**Affiliations:** 1 Minimal Access Therapy Training Unit (MATTU), Royal Surrey County Hospital NHS Foundation Trust, Guildford, Surrey GU2 7XX, UK; c.limb@surrey.ac.uk (C.L.); t.rockall@nhs.net (T.A.R.); 2 Division of Cancer, Department of Surgery and Cancer, Imperial College London, Hammersmith Hospital campus, Du Cane Road, London W12 0NN, UK; daniel.liu08@imperial.ac.uk (D.S.K.L.); e.rees@imperial.ac.uk (E.R.); j.krell@imperial.ac.uk (J.K.); 3 Omiics ApS, Åbogade 15, 8200 Aarhus N, Denmark; mtv@inano.au.dk; 4 Department of Histopathology, Royal Surrey County Hospital NHS Foundation Trust, Guildford, Surrey GU2 7XX, UK; izhar.bagwan@nhs.net; 5 Department of Medical Oncology, Amsterdam UMC VUmc, 1007 MB Amsterdam, The Netherlands; elisa.giovannetti@gmail.com; 6 Fondazione Pisana Per La Scienza, 56017 San Giuliano Terme PI, Italy; 7 Department of Clinical and Experimental Medicine, Faculty of Health and Medical Sciences, The Leggett Building, University of Surrey, Guildford, Surrey GU2 7WG, UK; h.pandha@surrey.ac.uk; 8 Department of General, Visceral, and Transplantation Surgery, University of Heidelberg, Im Neuenheimer Feld 110, 69120 Heidelberg, Germany; Oliver.Strobel@med.uni-heidelberg.de; 9 HPB Surgical Unit, Royal Surrey County Hospital NHS Foundation Trust, Guildford, Surrey GU2 7XX, UK

**Keywords:** biomarker, pancreatic cancer, pancreatic ductal adenocarcinoma, biliary tract cancer, circular RNA, circrna, circRNAs

## Abstract

**Simple Summary:**

Pancreatic and biliary tract cancers often present with non-specific symptoms, resulting in diagnosis at a late stage. This may be too late for curative surgery. Earlier detection and characterisation may guide treatment options and increase survival. Natural “circles” of RNA (circRNAs) are shown to regulate cancer-related genes, and act as cancer “biomarkers”. Recent research has shown that circRNAs are both abundant and stable, both of which are desirable characteristics for clinically useful biomarkers. In this systematic review, we describe the roles of circRNAs in pancreatic and biliary tract cancers, summarise the current published research and explore their utility as a biomarker. A total of 32 articles were included: 22 considering Pancreatic Cancer, 7 for Bile Duct Cancer and 3 for Gallbladder Cancer. CircRNA proved an exciting prospect as a biomarker for these cancers and future work should continue to develop and expand this field of research.

**Abstract:**

Pancreatic Ductal Adenocarcinoma (PDAC) and biliary-tract cancers (BTC) often present at a late stage, and consequently patients have poor survival-outcomes. Circular RNAs (circRNAs) are non-coding RNA molecules whose role in tumourigenesis has recently been realised. They are stable, conserved and abundant, with tissue-specific expression profiles. Therefore, significant interest has arisen in their use as potential biomarkers for PDAC and BTC. High-throughput methods and more advanced bioinformatic techniques have enabled better profiling and progressed our understanding of how circRNAs may function in the competing endogenous RNA (ceRNA) network to influence the transcriptome in these cancers. Therefore, the aim of this systematic review was to describe the roles of circRNAs in PDAC and BTC, their potential as biomarkers, and their function in the wider ceRNA network in regulating microRNAs and the transcriptome. Medline, Embase, Scopus and PubMed were systematically reviewed to identify all the studies addressing circRNAs in PDAC and BTC. A total of 32 articles were included: 22 considering PDAC, 7 for Cholangiocarcinoma (CCA) and 3 for Gallbladder Cancer (GBC). There were no studies investigating Ampullary Cancer. Dysregulated circRNA expression was associated with features of malignancy in vitro, in vivo, and ex vivo. Overall, there have been very few PDAC and BTC tissues profiled for circRNA signatures. Therefore, whilst the current studies have demonstrated some of their functions in these cancers, further work is required to elucidate their potential role as cancer biomarkers in tissue, biofluids and biopsies.

## 1. Narrative Review

### 1.1. Introduction

The incidence of pancreatic ductal adenocarcinoma (PDAC) has been increasing over the past three decades. Currently, almost 10,000 new cases are diagnosed each year in the UK, which is almost equal to the number of deaths related to PDAC [1]. In contrast to advances seen in many other cancers, the 5-year overall survival (OS) in PDAC has remained poor, with the most recent national data reporting ~5% 5-year survival in the UK, and 9% in the United States of America [1,2]. This is in part due to the late stage at presentation seen in 8 out of 10 patients, allowing an attempt at curative surgical intervention in only 20%. Similarly, patients with biliary tract cancers (BTC), such as cholangiocarcinoma (CCA), gallbladder (GBC) and ampullary carcinoma, have generally poor 5-year OS, at 5%, 17%, 21%, respectively [1,3]. Presentation of pancreaticobiliary malignancies is often non-specific, with features including abdominal and back pain, weight loss, fatigue and bloating. An earlier and more accurate diagnosis has the potential to identify these cancers at a lower stage, which would improve the opportunity for curative treatment, and increase survival. Therefore, the research community has been focused on identifying novel molecular biomarkers to improve diagnostic certainty, predict response to treatment, prognosticate, stratify, and develop targets for anti-cancer therapies.

The role of circRNAs has been demonstrated in various conditions, for example, diabetes [4] and Alzheimer’s disease [5], along with a number of cancers [6,7]. More recently their role in pancreaticobiliary cancers has been investigated, including PDAC, GBC and CCA. To date, there has been no research into circRNAs in ampullary cancer. In these tumours, differential expression of circRNAs may allow the discovery of several biomarkers. Developing an understanding of the role of circRNAs in these cancers and their regulation of gene expression, molecular pathways and protein production may also allow their utilisation as therapeutic targets. Expanding RNA profiling and array databases, deep sequencing techniques and evolving bioinformatics have allowed “in silico” mapping of circRNA competing endogenous RNA networks, attempting to hypothesise their in vivo mechanisms of action in various diseases.

In this narrative and systematic review, we describe the characteristics, biogenesis and biological mechanisms of action of circRNAs. We then perform a systematic review aiming to describe original research into candidate circRNA molecules: all studies quantifying and considering the clinical implications of circRNAs dysregulation in PDAC and other biliary malignancies will be included. This will be discussed in the context of the wider literature, with a focus on their demonstrated molecular mechanisms and potential clinical utilisation.

### 1.2. Circular RNAs

The vast majority of RNA molecules do not take part in protein translation and are therefore termed “non-coding” RNAs; this family accounts for 95% of the RNA pool [8,9]. This is a heterogeneous group including “short” molecules such as microRNAs (miRNAs), and small interfering RNAs; and “long” molecules termed long non-coding RNAs (lncRNAs), including circular RNAs (circRNAs). Much of the earlier work considering the role of non-coding RNA molecules focused on miRNA, such as lin-4 identified in the early 1990s [10], however, there has been increasing interest in circRNAs. CircRNAs were first found in viroids [11], and subsequently, hepatitis delta virus [12], before data demonstrated a widespread presence endogenous in human cells [13,14]. Next-generation sequencing approaches had been used for transcriptome studies (RNA-seq), but in the early days of RNA-seq these relied on mRNA enrichment by poly-A purification, which excludes circRNAs. Maturation of RNA-seq techniques to sequence non-poly-A RNA aided the detection of circRNAs. Early study of these molecules was limited by a lack of awareness as typical RNA amplification did not preserve circularity resulting in linear molecules, unidentifiable as circRNAs [15]. However, since these molecules were first recognised as single-stranded transcripts with a “scrambled” exon order [16], there has been increasing interest in their regulatory role in a number of human diseases with an increasing focus on cancer [17,18].

### 1.3. Characteristics

The inherent structure of circRNA results in a deficiency of 5′ to 3′ polarity and a lack of a polyadenylated tail [19]. This affords resistance against RNase R among other exoribonucleases and endoribonucleases [20], resulting in a relatively stable molecule. This circular structure has been shown to result in an extended half-life compared to its linear homologs, which can be over double in some described cases [21,22].

As circRNAs typically do not code for a protein molecule and initially had no clear function they were thought to be a by-product of typical post-transcriptional mRNA modification: formed by “mis-splicing” or indistinguishable from RNA lariats [23,24,25]. Despite this initial misconception, the majority of circRNAs have shown conservation across species, and it is now clear that they serve a number of genetic and molecular functions, and in some cases are found significantly more abundant than their linear counterparts [21]. Furthermore, they demonstrate tissue and development stage-dependent expression. For example, Memczak et al. described that a number of nematode circRNAs are specifically expressed in oocytes, but found to be absent in some cell embryos [15]. This provides evidence that circRNAs are not by-products of post-transcriptional mRNA processing, but that their production is a regulated process with specific molecular functions [13,21]. Further evidence of tissue-specific functionality is that dysregulation of specific circRNAs can result in both oncogenic and tumour suppressive features in a tissue-dependent manner [26]. It is now suggested that their inherent inertia and stability, including in response to stimulation from molecules such as EGFR [22], make functions related to longer-term processes, such as cell differentiation, more likely.

### 1.4. Biogenesis and Degradation

**Figure 1:**
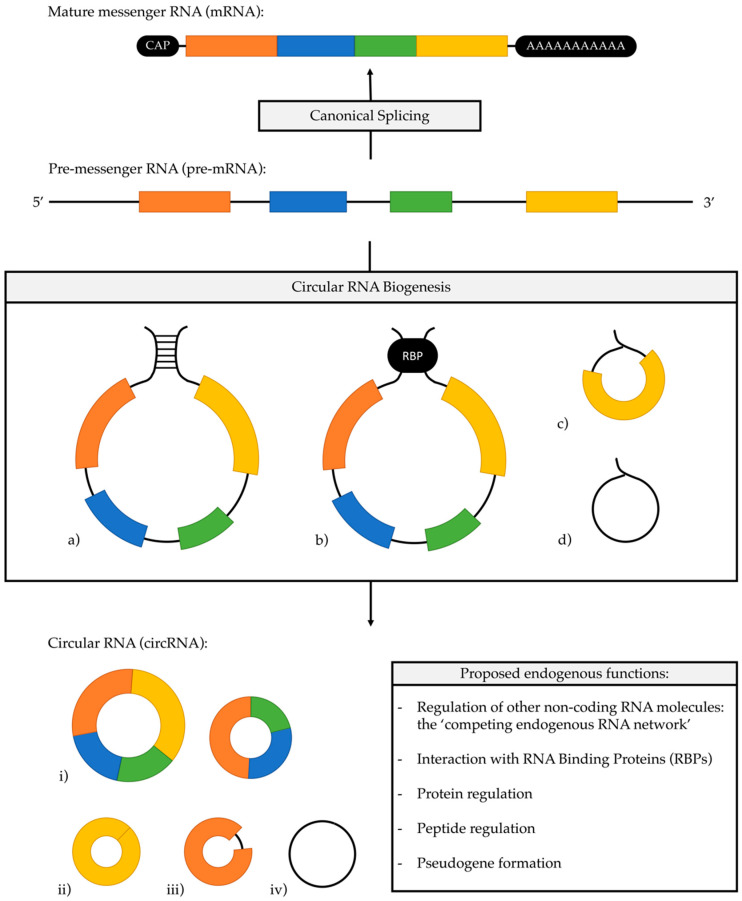
In typical canonical splicing (up), introns are excluded from immature pre-messenger RNA with covalent bonds forming between exons, the mature messenger RNA is completed with a 5′ cap and 3′ polyA tail; In circRNA biogenesis (down), a “back-splice” junction is formed between the 5′ splice donor site and an upstream 3′ acceptor site to form a circular molecule. Typical pathways include (**a**) reverse complementary nucleotide sequences in flanking introns, typically long Alu repeats, (**b**) RNA binding protein-mediated, and through lariat precursors, which may be a result of (**c**) exon skipping and (**d**) introns evading degradation; A variety of mature circRNA molecules can form from a single pre-messenger RNA sequence, including (**i**) multi-exonic, (**ii**) single exonic, (**iii**) exon-intronic, and (**iv**) intronic isoforms; Proposed endogenous functions are highlighted.

CircRNAs are formed during the post-transcriptional process of splicing pre-messenger RNA (pre-mRNA), where the canonical pathway typically excludes introns to form mature messenger RNA molecules as covalently linked exons. Back-splicing of pre-mRNA transcripts occurs with the formation of covalent bonds between a downstream 5′ splice donor site and an upstream 3′ acceptor site (Figure 1) [19,27]. One genetic location can generate both linear and circular RNAs, with data demonstrating a genetic correlation between linear and circular isoforms in 98% of cases [22]. Alternative splicing mechanisms have been shown to give rise to exonic (most common), intronic and intergenic circRNAs [28]. This process appears to be largely intron determined and to predominantly occur co-transcriptionally at the level of the gene [29,30]. Exon regions, or 5′ UTR sequences, are more prevalent in circRNAs, present in 80% of these molecules [31], and may occur as multi-exonic, single exonic and exon-intronic varieties [6].

The predominant mechanism for spliceosomal circRNA biogenesis relies on the presence of a reverse-complementary RNA sequence within introns flanking a potential circRNA [32]. Exons associated with circRNAs are commonly associated with long flanking intronic sequences and repetitive Alu elements, which are thought to facilitate circularisation by base-pairing and decreasing the distance between potential back-splicing sites [21,29]. Furthermore, the deletion of flaking Alu repeats has been shown to attenuation circRNA generation in vitro [33]. Alu repeats themselves pose an unpredictable mutagenic risk and enzymes such as DHX9 and adenosine deaminase (ADAR) play an important regulatory role in circRNAs biogenesis through destabilising intron pairing [34,35]. Alternative mechanisms in the literature for circRNA biogenesis include other intron-pairing motifs such as *quaking* (QKI) [36] and *muscleblind* (MBL/MBNL1) [29] or the involvement of exon containing lariat precursors [37].

As circRNAs lack 3′ and 5′ ends degradation, requires an initiating step of internal cleavage, for which a number of pathways have been identified. Primary sequence-dependent degradation can be Ago2/miR-671 mediated, as demonstrated in ciRS-7 (Cdr1as) [38], or mediated by an RNase P/MRP complex, which is able to act on m^6^A motifs after specific RBPs recruitment [39]. However, these mechanisms do not differentiate between linear and circular isoforms, and thus, they are not selective for circRNAs. Selective dysregulation of highly structured circRNAs has been shown to be mediated by the RBPs Regulator of nonsense transcripts 1 (UPF1) and Ras GTPase-activating protein-binding protein 1 (G3BP1), dependent on features of the 3′ untranslated region structure formed by specific base pairing [40]. Additionally, stimulation with Polyinosinic:polycytidylic acid and viral infection have both been shown to initiate RNase L mediated degradation, although the mechanisms remain unclear [41]. This controlled, and structure dependant degradation is essential for circRNA cellular homeostasis, supporting their importance in cellular function. Some evidence suggests a role for the discrete granular structures related to circRNAs, including P-bodies, Glutamate (GLU) bodies and stress granules, however, these require further research [42].

### 1.5. Biological Functions

#### 1.5.1. Competing Endogenous RNA Network

One of the principal mechanisms through which circRNAs have been proposed to regulate biological mechanisms is labelled “miRNA sponging”. This describes a regulatory axis between non-coding RNA molecules and mRNA termed the competing endogenous RNA (ceRNA) network [13,14,43]. MiRNAs are widely implicated, these small non-coding RNA molecules, 21–25 nucleotides long [44], act to regulate gene expression of mRNA. They function through mechanisms including molecular destabilisation, RNA cleavage and altered configuration, which reduces translational efficacy [45]. Through conserved segments termed miRNA response elements (MREs), circRNAs are able to interact with miRNA and attenuate their function, leading to a circRNA-miRNA-mRNA regulatory axis located in the cytoplasm [46,47,48]. Hansen et al. described over 70 conserved miR-7 target sites on ciRS-7 (Cdr1as) which allowed it to act as a competing endogenous RNA molecule and suppress the action of miR-7 [14]. This interaction is expected to be facilitated by other well-known RNA associated proteins involved in the RNA-induced silencing complex (RISC) such as Argonaute2 (AGO2) and this is the basis for AGO2 RNA immunoprecipitation (RIP) assays to examine circRNA and miRNA binding. However, the ceRNA hypothesis is not without controversy, with analysis of annotated circRNAs finding very few circRNA with more miRNA binding sites than expected by chance [49] and the levels of target sites required to demonstrate competing miRNAs may be exaggerated in some models to the extent that they are not physiologically relevant [50]. Furthermore, the function of circRNA is likely to be more complex, as an example the opposite effect to “sponging” has been found for circRNA ciRS-7 (Cdr1as), which has been reported to stabilise and transport miR-7, increasing its potency in suppressing related genes [51].

#### 1.5.2. Interaction with RNA-Binding Proteins (RBPs)

As mentioned, circRNAs can function as a direct miRNA sponge assisted by AGO2, one of the RBPs, but circRNAs can also act in an indirect way on mRNA transcription through RBPs, or compete with mRNA to combine with RBPs, to influence mRNA translation. Examples include circANRIL (circular isoform of the Antisense Non-coding RNA In the INK4 Locus), which was shown in vitro to control ribosomal RNA maturation by binding to a domain of Pescadillo Ribosomal Biogenesis Factor 1 (PES1), thereby preventing pre-rRNA binding and exonuclease-mediated rRNA maturation [52]. Other examples of RBPs shown to be influenced by circRNAs include Mannan-binding lectin [15], Rice Telomere-Binding Protein 1 (RTBP1) [53], Caspase-3 [54] and HuR [55]. It is also thought that circRNAs can associate with the U1 small nuclear ribonucleic protein (snRNP) in conjunction with RNA polymerase II in the nucleus, through which it is able to promote host gene transcription [15,56]. Furthermore, some varieties of circRNA are thought to influence gene expression through direct interaction with RNA polymerase II alone [56]. Intronic circRNAs in particular have little enrichment for miRNA target sites, lacking recognisable MREs, and so are not expected to have any role in protein production. The majority of these circRNAs accumulate in the nucleus, at sites of transcription, demonstrated through fluorescence in situ hybridisation (FISH) assays, and appear to promote genetic transcription with knockdown resulting in reduced expression of parental genes [23,57].

#### 1.5.3. Protein and Peptide Regulation

In the post-transcriptional setting, circRNAs share exons with linear isoforms. Spliceosomal mediated generation actively competes with canonical linear splicing. This may limit the production of linear homologs and has the potential to promote alternate “exon lacking” variants which may have attenuated or absent biological function [29]. For example, circRNAs that contain the translation initiation site may competitively sequester this from linear homologs and limit potential protein production, this has been termed the “mRNA trap” [58]. This may be of particular importance in cancer, where an imbalance in canonical and non-canonical splicing may influence the balance of key tumour suppressor and oncogene related proteins to promote tumourigenesis. This negative correlation requires further evaluation, and it should be noted that evidence suggests a positive correlation in most cases, likely as a result of upstream gene modulation [33].

The direct translation of circRNAs into proteins, however, remains controversial [59]. It has been demonstrated that a small number of circRNAs contain an internal ribosomal entry site (IRES), which allows the potential for translation [60,61]. There is mounting evidence to support circRNAs translation with a large number of “cap independent” protein coding sequences identified in the genome [15]. Circ-ZNF609 is an example that has been identified in eukaryote cells and proposed to translate into a protein. It contains a start-to-stop codon reading frame that could go through the process of translation, interestingly this complete sequence is generated through circularisation and so not present on related linear RNA molecules [62]. Additionally, ribo-circRNA UTR-dependant protein synthesis has been proposed and rolling circular amplification has been demonstrated in vitro [63]. M^6^A motifs are widely prevalent in circRNAs and likely to have a role in regulating protein synthesis, demonstrating the ability to enable and accelerate this process in vitro [64].

#### 1.5.4. Pseudogene Generation

CircRNAs have been proposed as a source of non-colinear pseudogenes (i.e., a circRNA-derived pseudogene would have an exon–exon junction in a reversed order) [63]. Retro-transcription and insertion into the host genome have the potential to alter the cellular genomic composition and subsequent gene expression. Although this potential function of circRNAs has been demonstrated within the human genome, the biological relevance and role remain unclear.

### 1.6. CircRNA Research Techniques

#### 1.6.1. CircRNA Sequencing and Profiling

One of the biggest challenges in the study of circRNAs has been identification and differentiation from other non-coding RNA molecules. Early recognition has been driven through identification of the “back-spliced” sequences [16]. One limitation of this technique, however, is inappropriate identification of an apparent back-spliced sequence, generated through another cellular mechanism such as reverse transcriptase template switching or tandem DNA duplication [58]. Specific approaches to differentiate these such as targeting enzymatic degradation via the 3′ polyadenylation tail and gel electrophoresis, augmented by increased cross-linking may allow more accurate assessment. In addition, weak hydrolysis and targeted RNase H degradation can allow the linearization of circRNAs for further evaluation.

CircRNA profiling can be broadly divided into two techniques: tissue microarray or RNA sequencing. Tissue microarray is a high throughput technique in which specific circRNA junction sequences are identified to quantify expression. The Arraystar human CircRNA array (Arraystar^TM^) investigates for 13,617 circRNAs and was utilised by two studies comparing circRNAs expression in PDAC and para-cancerous tissue. These two projects identified 351 (fold change of >1.5) and 289 (fold change of 2) dysregulated circRNAs expression profiles respectively as potential targets for future investigation. The results were submitted to the Gene Expression Omnibus under numbers GSE69362 (6 paired samples) [65] and GSE79634 (20 paired samples) [66]. Other probe-based platforms, for example, NanoString, have been utilised by researchers to investigate other malignancies, however, are currently limited to custom-made plates with a small number of circRNAs targets. As microarray and other probe-based detection methods identify specific predefined back-spliced sequences, identification and quantification of specific circRNAs are good, however, circRNAs that are not included in the target dataset will be ignored. Deeper RNA sequencing is able to describe the wider landscape of RNA expression and each study has the potential to identify novel circRNAs that are dysregulated in PDAC [67], but is not without limitations in the circRNA field. Problems include technical artefacts leading to false positives, difficulties with maintaining high algorithmic sensitivity with low read counts in computational workflows, the use of initial RNase fragmentation, and complex data output which requires significant computational and bioinformatics analysis [68].

After identification of specific candidate circRNAs, the expression can be quantified via Real-Time quantitative Polymerase Chain Reaction (RT-qPCR). To achieve this, outward-facing primers are utilised with a complementary nucleotide sequence to the “back-splice” [69]. This is relatively inexpensive, however false high readings be generated where multiple isomers exist for a single back-splice event [70]. Sanger sequencing can also be utilised in experimental validation, and allows differentiation between species of circRNA isoforms. Quantification after treatment with RNAse R can be used to demonstrate resistance to degradation, supporting evidence of circularity in candidate molecules [20].

RNA research techniques can be limited by RNA degradation, although meticulous handling in an RNase-free environment and minimisation of pre-analytical time from sample to bench can limit this to an extent. This impact may be further attenuated when working with circRNAs, due to their increased stability, and resistance to enzymatic degradation. Currently, the majority of circRNAs research utilises fresh frozen tissue samples, although some researchers have considered the role of alternate tissue sources. Indeed, one study considered the potential of fresh frozen paraffin-embedded (FFPE) tissue samples [71]. This study compared FFPE and fresh tissues, finding a good correlation of circRNA expression between each type, and that differential circRNAs were able to positively identify tumour samples in both cases. Of note, in this study, fresh tissue samples demonstrated a higher number of differentially expressed circRNAs. This is important, as although fresh frozen tissue samples may currently appear to be the most effective approach, collecting a large number of samples (especially those with specific features) is not always feasible, and so efforts to characterise circRNAs in archived FFPE samples may hold potential value.

#### 1.6.2. Bioinformatics

The ability for researchers to identify potential ceRNA networks through complementary binding sites has advanced significantly with the expansion of publicly available sequence databases and bioinformatic technology. CircInteractome (https://circinteractome.nia.nih.gov) is one web tool that has been developed to explore circRNA structure and potential interaction with RNA binding proteins (RBPs) and miRNA within the ceRNA network [61]. This tool takes data from a number of different sources, including RBP binding sites identified by cross-linking immunoprecipitation (CLIP) techniques and potential miRNA target sequences incorporated in the TargetScan algorithm, to map potential circRNAs interactions. Interestingly this had demonstrated significantly more complementary binding sites for both RBPs and miRNA than would be expected through chance, supporting the ceRNA theory. This is one technique through which RBPs, such as Quaking and DHX9, have been hypothesised to have a regulatory role in circRNA expression [34,72].

Other tools commonly used in the understanding of the ceRNA network include StarBase (for RBP interactions) and Arraystar’s homemade miRNA prediction software, miRanda and StarBase (for miRNA and mRNA interactions) [73]. Identification of dysregulated circRNAs has allowed studies to utilise techniques such as dual-luciferase reporter assays to validate these relationships in vitro. In addition to mapping ceRNA networks, the progression of this technology in web tools such as CircInteractome has advanced research techniques by facilitating the development of junction primers for specific circRNA identification and designing specific siRNA molecules able to competitively inhibit and silence circRNA function.

Publicly available datasets such as Gene Ontology (GO) and the Kyoto Encyclopaedia of Genes and Genomes (KEGG) allow evaluation of potential interplay between ceRNA networks and known genetic and molecular pathways [65,74,75]. GO analysis can be used to hypothesis the enrichment of ceRNA networks for features such as RNA splicing, cell cycle and cell signalling [75]. Association of circRNAs with specific biological functions, in particular those that may be related to features of malignancy, may help to understand the clinical impact of dysregulation. In addition, GO analysis has allowed the identification of a wide panel of transcription factors, including the oncogenes p53 and MYC, that may be implicated in the regulation of circRNAs [75]. KEGG analysis allows consideration of potential influence on molecular and signalling pathways such as WNT and autophagy and their related protein molecules, which again may help to develop an understanding of their role in cancer [76]. The visualisation of these relationships can be integrated and conceptualised through software such as Cytoscape [77].

## 2. Results

### 2.1. Studies Included

**Figure 2:**
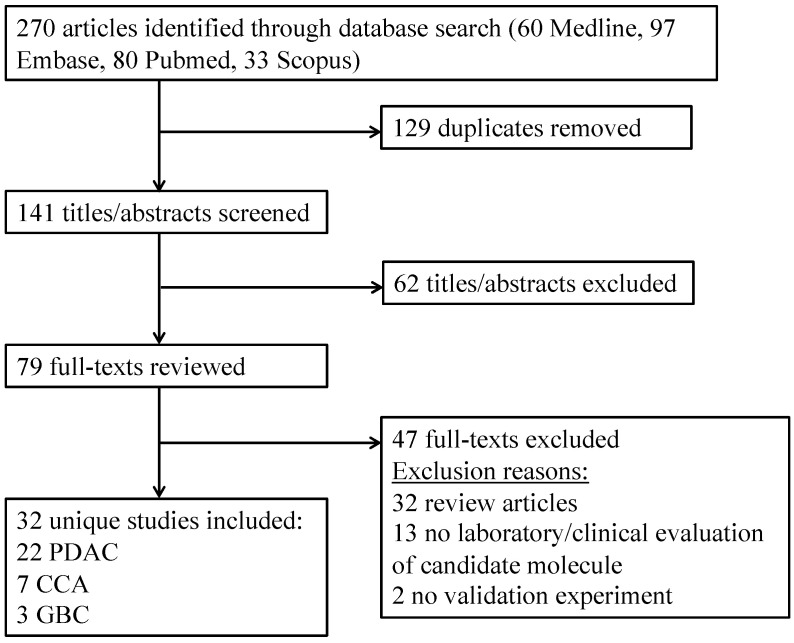
Flow-diagram for study selection.

The defined search strategy identified 270 articles of which 129 duplicates were excluded. At title/abstract screening 62 articles were excluded as clearly unrelated to the study question or excluded article type. On full-text review, a further 47 articles were excluded that did not present unique data with adequate candidate evaluation. No additional studies were identified through the reference review. Therefore, 32 articles were included in this systematic review: 22 PDAC, 7 CCA and 3 GBC. This is summarised in Figure 2.

### 2.2. Circular RNA Expression

Twenty studies validating circRNAs expression in PDAC were included, all of which utilised real-time quantitative polymerase chain reaction (RT-qPCR) to evaluate (Table 1). A total of 22 circRNAs were evaluated of which 19 were upregulated and 3 downregulated.

Bioinformatic techniques were utilised by 20 groups to evaluate the relationship of circRNAs molecules in the ceRNA network, demonstrating a number of complementary miRNA binding sites to hypothesis regulatory roles over molecules and molecular pathways (Table 2).

### 2.3. In Vitro and In Vivo Characteristics

CircRNA expression in PDAC cell lines generally correlates with the differential expression demonstrated in tissue samples. Furthermore, cell studies have allowed quantification of the impact of differentially expressed circRNAs with cellular features of malignancy (i.e., proliferation and colony formation, invasion and migration and apoptosis) (Table 3). In those circRNAs found to be overexpressed in PDAC, ectopic or stimulated overexpression generally enhances features of malignancy such as proliferation, viability, migration and invasion whilst inhibiting apoptosis; while silencing and reducing expression attenuates those features of malignancy. In one study, this was supported through measuring markers of proliferation (i.e., c-Myc and cyclin D1), along with markers of invasion and metastasis (i.e., vimentin and E-cadherin) [94]. Conversely, where under expression has been associated with malignancy, ectopic expression has been shown to inhibit colony-forming ability and proliferation, whilst promoting apoptosis. Co-transfection rescue experiments were performed, and returning circRNA expression to control levels was universally shown to reverse this effect. One study found that silencing hsa_circ_001653, demonstrated to be upregulated in PDAC, resulted in decreased angiogenic capacity, vascular length and the number of vascular branches; with overexpression giving the opposite results [48]. Overexpression of hsa_circ_001587, shown to be downregulated in PDAC, resulted in reduced tube formation suggesting inhibition of angiogenic capacity [95]. Silencing had the opposite effect.

Animal studies have demonstrated that PDAC cells in which implicated circRNAs are overexpressed are associated with increased tumour size and evidence of metastatic disease, while silencing results in a reduction of tumour size and growth (Table 4).

### 2.4. Clinical Disease Characteristics

Given the potential mechanisms and cellular effects that are described, it is not surprising to find that the dysregulation of implicated circRNAs in PDAC is associated with poor prognostic features and ultimately shortened survival time (Table 5). No association has been demonstrated between circRNAs expression and demographics such as age, sex and tumour location in any study. No association has been found with carcinoembryonic antigen (CEA); one study found a positive correlation with Carbohydrate antigen 19-9 (CA 19-9) [79]. Dysregulation of investigated circRNAs has been most closely associated with lymphatic and vascular invasion, metastatic disease, decrease cellular differentiation, duodenal invasion and stage. It must be noted that it is not clear from the current data whether abnormal circRNAs expression is a cause or result of advancing disease, although in some examples multivariate analysis has suggested it is an independent prognostic factor for reduced overall and disease-free survival [92,96]. Interestingly, in some studies, circRNA dysregulation is more strongly correlated than all other clinical features, including stage and lymphatic spread, with reduced overall and disease-free survival at multivariate analysis [84,93].

### 2.5. Results for Biliary Tract Malignancies

Less work has been undertaken to explore the role of circRNAs in other biliary malignancies, although studies investigating both CCA and GBC have been published. A total of 7 studies considering CCA, and 3 considering GBC were identified (Table 6).

Within CCA, the circRNA hsa_circ_0005230 has been demonstrated to be upregulated in cancer tissue compared to control, and correlated with increased tumour size, lymphatic spread and disease stage [97]. Its action was proposed to be through sponging miR-1238 and miR-1299, validated through dual-luciferase reporting assay. Silencing reduced the malignant features, including proliferation, migration and invasion, while increasing apoptosis in cell studies; ectopic expression to cells had the opposite effect. In animal studies, hsa_circ_0005230 downregulation reduced metastatic deposits. This same group demonstrated that hsa_circ_0001649 was conversely downregulated in CCA compared to control [98]. Although the mechanism for this was not clear, downregulation was associated with increased tumour size and decrease cellular differentiation. In cellular studies circRNAs silencing resulted in increased proliferation, colony formation and migration with increased apoptosis, overexpression had the opposite effect. In animal studies overexpression was able to reduce tumour size. The expression of ciRS-7 (Cdr1as), which is known to interact with miR-7, has been demonstrated to be unregulated in CCA and is positively associated with lymphatic spread, stage and recurrence along with reduced survival time [96]. Further work demonstrated its association with the features of malignancy in vitro and in vivo [99]. This study however suggested miR-641 as an additionally clinically relevant target, reporting 10 complementary binding sites after bioinformatical analysis and proposing its function through action on the AKT/mTOR pathway. The expression of hsa_circ_0000284 has been found to be upregulated in CCA tissue and exosomes [100]. This molecule was found to be associated with proliferation, migration and invasion in CCA cell lines and knockdown resulted in reduced tumour size and metastatic disease in vivo. It is proposed to function through modulation of the mir-637/LY6E regulatory axis. Furthermore, this work proposed that exosomes were a critical method for hsa_circ_0000284 dissemination, found to be secreted from CCA cells and to induce features of migration and proliferation in local normal cells. A large study recently identified the upregulation of hsa_circ_102064 in CCA tissue and in extracellular vesicles (EVs) from both serum and bile [101]. The upstream gene for the circRNA in ERBB2, and alternative circRNA from this site has previously been implicated in the gallbladder and gastric cancer [33,102] and so this was named cholangiocarcinoma associated circular RNA 1 (circ-CCAC1) by the authors. Serum dysregulation was broadly associated with an increased number of tumours, lymph node metastasis and advanced TNM stage. Furthermore, hsa_circ_102064 was associated with vascular invasion in perihilar CCA; tumour size in distal CCA; and poorer prognosis and recurrence in intrahepatic CCA, in which upregulation was also an independent prognostic marker. Silencing resulted in reduced features of proliferation, migration and invasion in vitro. Depletion attenuated xenograft size and metastatic disease in vivo. Hsa_circ_102064 demonstrated the ability to enter cells via extracellular vesicles (EVs) where it resulted in increased vascular permeability and induced angiogenesis. This circRNA was proposed to act through sponging of miR-514a-5p, a predominantly cytoplasmic miRNA, resulting in the upregulation of YY1 and activation of its downstream transcription factor CAMLG. It was also demonstrated to sequester the RBP E2H2, increasing vascular permeability through modulating SH3GL2/ZO-1/Occludin signalling. Throughout these studies, CCA location within the biliary tract was not statistically associated with candidate circRNAs expression.

With regards to GBC, the circRNA hsa_circ_0008234, or circFOXP1, demonstrated increased expression in cancer compared to controls [53]. Higher expression was associated with the lymphatic spread and TNM stage. High expression was an independent risk factor for reduced survival. Increased expression was associated with increased proliferation, invasion and migration, shortened cell cycle and reduced apoptosis in cell studies; and increased tumour growth in animal studies. The opposite findings were demonstrated after silencing. This was suggested to be dependent on its regulatory “sponge” function of miR-370. This controls the development of the protein PKLR, and this relationship was validated through the dual-luciferase reporting assay. PKLR appears to have a vital role in the Warburg effect, aerobic glycolysis which drives cellular proliferation, invasion and migration in cancers such as this. Hsa_circ_0008234 knockdown resulting in its features of reduced lactate, pyruvate and extracellular acidosis with increased oxygen consumption. In another study, hsa_circ_0000284 was demonstrated to be overexpressed in two patient-derived cell lines compared to controls [26]. Silencing resulted in reduced proliferation and colony-forming ability with increased apoptosis; transfection to increase expression has the opposite effect. This is proposed to act through its interaction with miR-124, known to act on ROCK1 (rho-associated protein kinase 1) and CDK6. Finally, circERBB2 demonstrated upregulation in GBC tissue and a statistical association between overexpression and shorter survival was identified [33]. Laboratory study demonstrated the suppression of circERBB2 to attenuate the proliferation of GBC cell lines and reduce tumour size on mouse xenografts. Although a number of miRNA binding sites were identified in this molecule these were of small frequency and not clearly associated with tumour related molecular pathways. This team proposes that circERBB2 acts through regulating RNA polymerase I mediated ribosome 45 s synthesis at the nucleus, a characteristic rate-limiting step on tumorigenesis. CircERBB2 was found to be enriched in the nucleus and appeared to regulate nucleolar localisation of PA2G4, a key RNA-binding protein involved in ribosomal assembly.

## 3. Discussion

### 3.1. Biological Role of circRNA in PDAC

Molecular pathways and proteins that are associated with the development of the malignant features in PDAC are demonstrated to be part of dysregulated circRNAs ceRNA networks (Table 2). Various mechanisms are proposed for their function in driving carcinogenesis, predominantly through modification of the local tumour microenvironment. These include promoting dysregulation of the cell cycle, enhancing migratory and invasive capacity, attenuating the immune response, promoting endothelial-mesenchymal translation (EMT) and enabling resistance to chemotherapy.

#### 3.1.1. Increasing Cell Proliferation

Enhanced proliferation can be achieved through a number of mechanisms, including resistance to apoptosis. A number of molecular pathways regulate the cell cycle including EGFR/STAT and PI_3_K/AKT, with evidence that these can be influenced by circRNAs. EGFR is an upstream regulator and activator of a number of pathways associated with the development of malignancy, including the Signal Transducers and Activators of Transcription (STATs). Activated STAT may then stimulate specific oncogenes, leading to the development of malignancy. CircRNAs are shown to have a regulatory role, for example, ciRS-7 (Cdr1as) is able to promote the EGFR/STAT3 pathway through suppression of miR-7 in the ceRNA network [85]. In the PI_3_K/AKT pathway, PI_3_K is activated through ligand binding, which phosphorylates AKT, again promoting cellular proliferation. This pathway has been implicated in the growth and progression of PDAC with emerging evidence that circRNAs play a role in its regulation [74]. HOXC6, previously implicated in both breast and prostate cancer, has an important role in regulating cellular development, including differentiation, apoptosis, signalling and subsequent angiogenesis. Hsa_circ_001653 has been proposed to have a regulatory role over this protein through the ceRNA network, interacting with miR-377 and AGO2, demonstrating the ability to influence biological activity in vitro [48].

#### 3.1.2. Enhancing Tumour Invasion and Metastasis

The integrity of the cellular micro-environment, including cell–cell interactions and extracellular matrix, is key to homeostasis. Both interruption of the endothelial barrier and disruption of the extracellular membrane permit cellular dissemination, synonymous with the features of invasion and metastasis in malignancy. Circ-IRAS has been implicated in the development of tumour invasion and metastasis through attenuating the cell tight junction barrier. Attenuation of miR-122 within the ceRNA network results in increased RhoA activity with subsequent increase in F-actin and decrease ZO-1 tight membrane proteins. This results in increased endothelial monolayer permeability [82].

In addition to acting through the “miRNA sponge” studies have associated circRNA protein binding with the progression of malignancy, for example between circFOXK2 and both YBX1 and hnRNPK [91]. The formation of this complex promotes the increased activity of the oncogenes NUF2 and PDXK in vitro. This increases the invasive and metastatic potential of PDAC cells through attenuation of cell adhesion and dysregulation of mRNA splicing. Other proposed protein interactions include the extracellular matrix protein MMP7, which plays an active role in extracellular matrix degradation [86,98]. Furthermore, local desmoplasia is a critical feature in PDAC and may be driven by proteins that can form complexes with circRNAs such as COL1A1 [86]. These mechanisms are also relevant in the context of exosomes, which may contain oncogenic circRNAs.

#### 3.1.3. Promoting Angiogenesis and Lymphangiogenesis

Angiogenesis is critical in both physiological and pathological states [104]. It is a route of nutritional support, which may include oxygenation, along with the removal of waste products, for example, carbon dioxide. Overexpression of vascular angiogenic factors, such as vascular endothelial growth factor A (VEGFA), is associated with rapid growth in tumour cells [105]. Pan-cancer analysis has demonstrated the circRNA ciRS-7 (Cdr1as) is correlated with increased cancer-associated endothelial cells coupled with pathological angiogenesis [106]. Within PDAC, overexpression of Circ-ASH2L has been proposed to stimulate angiogenesis through the upregulation of the Notch 1 pathway, in turn stimulating downstream VEGFa activity [88]. CircADAM9 has also been proposed to modulate VEGF activity through attenuating the inhibitory effect of miR-217 on serine protease 3 (PRSS3) [89]. This oncogene is associated with advanced features in PDAC [107] and is thought to affect through ERK/VEGF stimulation. Additionally, EGFR may also have a role in ERK related pathways; it is able to phosphorylate MAPK via tyrosine kinase to promote ERK activation [108].

Lymph node dissemination, enhanced by lymphangiogenesis, is one pathway though to be responsible for rapid spread and metastasis in PDAC [109]. Current research suggests that downregulation of hsa_circ_0086375 (circNFIB1), shown to be downregulated in PDAC, may support this mechanism. In vitro suppression of hsa_circ_0086375 resulted in increased tube formation and migratory capacity of PDAC cells, with the converse true after overexpression [93]. Furthermore, this study demonstrated that hsa_circ_0086375 expression was negatively associated with lymphatic vessel density and was lower in metastatic tumour cells in the lymph nodes, when compared to PDAC tissue, suggesting this circRNA may enable migration of tumour cells.

#### 3.1.4. Attenuating the Immune Response

Both CircUBAP and hsa_circ_0000977 have been hypothesised to promote malignancy in PDAC through attenuation of the host immune system [74,110]. CircUBAP is able to bind and inhibit miR-494, which in turn attenuates its action on CXCR4 and ZEB1. This is proposed to result in preventing T cell tumour infiltration and limiting antigen presentation, resulting in impaired tumour recognition and therefore enabling tumour escape mechanisms. Alternatively, hsa_circ_0000977 was proposed to facilitate immune escape by limiting Natural Killer (NK) cell activity. Hsa_circ_000977 was shown to be upregulated in the presence of hypoxia, and through miR-153 sponging able to modulate HIF1A mediated immune escape through upregulation of HIF1A and ADAM10.

#### 3.1.5. Epithelial to Mesenchymal Transition and Cancer Stem Cells

Current research has demonstrated a relationship between circRNAs and the process of epithelial to mesenchymal transition (EMT). Definable, and typically increased, regulation of many circRNAs has been demonstrated in cells that have been induced to go through this process [36]. EMT is characterised by a loss of cellular polarity and impaired cell–cell interactions, resulting in the development of migratory and invasive cellular characteristics, key features of malignancy. The transforming growth factor β (TGF-β) growth factor family is considered the main inducer of EMT, and one typical signalling pathway includes the phosphorylation and activation of the SMAD (Small body size and Mothers Against Decapentaplegic) related proteins [111]. EMT is regulated by a number of mechanisms including transcription factors, for example, the Zinc finger proteins SNAIL and ZEB1 (Zinc finger E-box-binding homeobox 1), and more recently microRNA molecules, for example, miR-200 and miR-34 [111]. In vitro results now support a relationship between dysregulated circRNAs and EMT, such as circHIPK3 and hsa_circ_0013912, along with downregulation of epithelial markers, such as E-cadherin [90,94].

Identification of cancer stem cells (CSCs) can be achieved by evaluation for surface markers, such as CD44 and CD133, via immunostaining, or by performing sphere formation assays and in vivo tumour initiation assays. CircRNAs have been shown to maintain CSCs through a variety of mechanisms, such as WNT and Notch signalling regulation [112]. CircFOXP1, for example, is able to promote tumorigenesis and stem cell phenotype through modulation of EFGR and WNT pathways, via sponging miR-17-3p and miR-127-5p [113]. Both in vitro and in vivo studies have supported this hypothesis, finding that the silencing of circFOXP1 impaired cellular differentiation. These features are strongly associated with resistance to chemotherapeutic agents. In bladder and lung cancer, circRNAs have been shown to promote self-renewal which is a typical property of CSCs, and some candidates, such as circASXL1 and circHIPK3, have been proposed as markers of CSCs [63].

#### 3.1.6. Chemotherapy Resistance

Gemcitabine resistance is one of the principal challenges in providing effecting chemotherapy for advanced PDAC. Although the underlying mechanisms remain unclear, previous work has demonstrated that overexpression of some members of the ceRNA network is associated with chemotherapy resistance in PDAC, driving the loss of miR-410-3p [114]. More recent data have implicated the differential expression of specific circRNAs in chemotherapy-resistant PDAC and CCA cells, including ciRS-7, circHIPK3 and circSMARCA5 [85,90,103]. The expression profile of circRNAs between PDAC cell lines, with and without gemcitabine resistance, has been shown to differ significantly [115,116]. Furthermore, for the two most dysregulated candidates, this dysregulation was shown to persist when comparing plasma samples from patients, and was able to predict clinical response to gemcitabine compared to non-responders [116]. Cells overexpressing ciRS-7 (CDR1as) are thought to demonstrate increased gemcitabine resistance as a result of impaired regulation of EGFR/STAT3 signalling pathway [85]. Conversely, the downregulation of circ_SMARCA5 is associated with increased gemcitabine/cis-platin resistance in CCA [103]. The function of its host gene, SMARCA5, includes protecting the DNA against damage and DNA repair; and so, a reduction in circ_SMARCA5 has been postulated to represent a loss of this function. The upregulation of circHIPK3 is evident in gemcitabine resistance PDAC cell lines [90]. This is proposed to negatively regulate RASSF1 through sponging miR-330-5p; knockdown results in attenuation of the features of malignancy.

### 3.2. Clinical Utilisations of circRNAs

These studies clearly demonstrate significant differential expression of certain circRNAs molecules in pancreatic and biliary tract tumours compared to normal tissues. Furthermore, this differential expression can persist in both serum and circulating exosomes. Both in vivo and in vitro, specific circRNA expression has been associated with features of malignancy. These data, along with increasing evidence that these molecules are abundant and stable, demonstrate the significant potential for utilisation of circRNAs as biomarkers in PDAC and BTC as diagnostics and prognostics, with the potential for developing future predictive and therapeutic applications. Therapeutic applications would be related to their individual biological roles, including their relationship with the features of malignancy, and any association with the development of chemotherapeutic resistance.

#### 3.2.1. Diagnostic Biomarkers

Consideration of circRNAs as biomarkers has been undertaken in a variety of gastrointestinal cancers. One meta-analysis included 13 cancer studies: 7 gastric, 5 hepatocellular and 1 colorectal, demonstrating an area under the Receiver Operating Curve (AUC) of 0.81 for single circRNA candidates, with a corresponding sensitivity of 0.72, and specificity of 0.77 [117]. Its positive likelihood was 3.09, and negative likelihood 0.37 with a diagnostic odds ratio of 8.38. Hsa_circ_0006988 has been specifically investigated as a candidate blood-based biomarker in PDAC, demonstrating an AUC of 0.67 [79]. However, the results from this study demonstrated a lower sensitivity and specificity of this circRNA molecule, compared to the standard biomarker, carbohydrate associated Antigen (CA) 19-9. Interestingly, the AUC in combination with CA 19-9 was greater than either biomarker alone. This highlights the importance of a multimodal approach to diagnostics, suggesting that circRNAs may be useful for investigating patients lacking the Lewis antigen, with A- B- blood types, in whom CA 19-9 will not be elevated. Furthermore, in addition to considering circRNA expression alongside traditional tumour markers, one group has demonstrated that the diagnostic value of circRNAs can be improved when assessing combinatorial circRNA expression [118]. This was shown in gastric cancer, where it was demonstrated that a pooled AUC of 0.97, with a sensitivity of 0.89 and a specificity of 0.94 could be achieved. The diagnostic performance improved significantly when using a circRNA “signature” expression, compared to a single circRNA candidate, and the authors concluded that a pooled expression has better performance and potential for cancer diagnostics. This avenue is yet to be explored in PDAC.

One limitation when using tissue for diagnostics is the invasive nature of sample collection, and this is typically obtained at the time of surgery when the diagnosis is already clear. This has led to the consideration of other potential sites of interest, including saliva, shown to be relevant in other malignancies [119], both cyst fluid and cytology obtained during fine needle aspiration (FNA) and plasma/serum samples. There has been no research in the context of PDAC investigating circRNAs expression in saliva samples, nor those obtained at FNA, however, there has been interest in the potential for plasma or serum samples to be utilised. CircRNAs have been demonstrated to be enriched in plasma samples for PDAC [15], and in other malignancies, they have been demonstrated to have a reliable ability for diagnosis [118], and there has also been promising work investigating the role of circRNAs in exosomes and other EVs.

Both plasma and serum are easy to isolate from peripheral blood samples, with defined standard operating procedures [120]. Two studies investigating plasma circRNAs expression in PDAC have demonstrated a demonstrating significant, and correlative, dysregulation of candidate circRNAs in both tissue and plasma samples [79,116]. This highlights the potential of plasma or serum measurement of circRNAs, however, important methodological information was lacking, and neither of these studies reported the quality or quantity of total RNA extracted from each blood sample for analysis, or their techniques for validation.

Extracellular vesicles (EVs) are a compartment that is received increasing attention. Exosomes are 40–150nm sized EVs generated from the plasma membrane of cells as part of the endosome pathway [121]. These structures are formed of a lipid bilayer and found present in the systemic circulation. They are one means of communication between tumour cells and their microenvironment, including endothelial and immune cells, and can regulate tumour genesis and malignancy through an effect on angiogenesis, inflammation and the host immune response. Exosomes have the ability to both contain and deliver genetic information [122]. It has been demonstrated that circRNAs are abundant in exosomes, with a 2–6 times higher circ-linear RNA ratio, for some examples, than found in cells; and exosomes containing circRNAs have demonstrated the ability to pass through PDAC cell walls in vitro demonstrating this mechanism for circRNAs release [47,123]. Increased circRNAs abundance could be a passive result of their increased degradation time, however, it has been suggested that active enrichment of exosomal circRNAs may also occur. Exosomal circRNAs are not only abundant, but also independent of the intracellular expression [124]. Thus, cellular exosomal release can be both active and passive, and in some cases attenuated by molecular pathways associated with differentially expressed circRNAs [82].

The profile of exosomal circRNAs expression has been described in one study that sequenced RNA in 14 serum samples (GSE100232) [125]. This study describes abundant circRNAs in circulating exosomes, along with other RNA molecules, and highlights the potential of circRNAs as novel biomarkers. It did note that circRNA expression was of varied distribution, and of low expression in some cases. Furthermore, the small exosomal volume in serum, and therefore the limited quantity of circRNAs are challenges for circulating circRNA evaluation. These results were incorporated into a proactively updated web database, called exoRBase, which describes 58,330 circRNAs sequences, along with lncRNA and miRNA, from this study, as well as sequencing data for other diseases [126]. Considering specific examples, tumour exosomes containing an increased volume of circ-PDE8A and circ-IRAS were identified in the systemic circulation of PDAC patients. These circRNAs are overexpressed in PDAC tissue, and have been associated with disease progression, reduced survival and metastatic disease [47,82]. Exosomal circRNA expression profiles are altered between PDAC patients and healthy controls and have been validated in PDAC cells [127]. As well as the potential ability to transport circRNAs between cells locoregionally, it has been suggested that circRNAs in exosomes may act as a vehicle for miRNA, which may then suppress corresponding gene expression at distant sites [128].

#### 3.2.2. Prognostic and Predictive Biomarkers

Further to acting as a diagnostic biomarkers, plasma/serum circRNAs level may allow prognostication of disease and prediction of response to treatments. Thus, dysregulated peripheral blood circRNA expression has been strongly correlated with tumour factors and survival outcomes in PDAC, and unlike pathological evaluation, can allow a non-invasive, real-time, assessment of the tumour transcriptome. This “liquid biopsy” has the potential to allow proactive monitoring of the disease process, permitting improved recognition of treatment success or failure, and act as a step towards tailor-made treatments [129]. Regular measurement of circRNA expression may offer utility as an adjunct during follow-up after surgical resection, with the potential to improve early recognition of recurrent disease. CircRNAs, in particular, have potential as clinically useful blood-based biomarkers, as their resistance to degradation may allow detection in the context of high circulating RNase in PDAC [130].

With curative surgical intervention possible in only a limited number of PDAC patients [1], primary chemotherapy is now being investigated. Although chemotherapy improves survival time in PDAC, its use is limited as cancer develops resistance to the agents used [131]. Combinations of chemotherapeutic agents have offered limited value with significant associated morbidity [132]. As described a number of circRNAs have been associated with chemotherapy resistance in PDAC and BTC [85,90,103]. Furthermore, in addition to potentially acting as biomarkers to predict a patient’s response to chemotherapy, in vitro experiments have raised the possible role of circRNAs as therapeutic targets for anti-cancer treatments. In the above studies, silencing of implicated circRNAs was able to generate sensitivity to chemotherapy in previously resistant PDAC cell lines, and overexpression of downregulated circRNAs was able to increase chemotherapy sensitivity in resistant CCA cell lines [85,103]. However, these findings were not consistent across all the cell lines investigated.

#### 3.2.3. CircRNAs as Therapeutic Targets

Some circRNAs, such as hsa_circ_0001649, have been demonstrated to generate tumour suppressive effects, and these offer potential as therapeutic targets [83]. Stimulated overexpression reduced proliferation and colony-forming ability, while increasing apoptosis in PDAC, CCA and GBC cell lines (see Table 3 and Table 6). Furthermore, circRNAs have been suggested to have a regulatory role over angiogenesis, lymphangiogenesis and immune function. A number of circRNAs appear to regulate EMT and progression of CSCs, which are both critical to supporting the maintenance of “stemness” [112]. Furthermore, “stemness” appears to be related to gemcitabine resistance [133]. CircRNA dysregulation is seen in gemcitabine resistance, and silencing of implicated circRNAs was able to restore gemcitabine sensitivity in a resistance cell line [116]. With an appropriate technique for induction, manipulating circRNAs in vivo may be able to influence response to chemotherapeutics for these tumours.

Exosomes and other EVs have been suggested as a possible delivery vehicle for anti-cancer therapy. In animal models, exosomes have been used to increase the therapeutic index of doxorubicin [134], and their previously described ability to transport non-coding RNA molecules [121] may be a novel therapeutic strategy for delivering cancer-suppressing circRNAs. In addition, several in vitro studies have shown that siRNA molecules have the potential to be delivered using exosomes in order to silence tumour promoting circRNAs. One group injected PDAC cell lines into nude mice after siRNA silencing of an upregulated circRNA: this resulted in attenuated tumour development, with a consequent reduction in tumour size. Conversely forced overexpression of hsa_circ_0001649, which has reduced expression in CCA, resulted in a similar tumour size reduction effect in animal studies [98]. These data demonstrate that circRNAs have the potential as therapeutic targets, and the development of tools such as CircInteractome [61] has improved the ability to develop siRNAs able to selectively silence circRNAs of interest.

### 3.3. Future Research

At present, the circRNA component of the transcriptome has only been assessed in a limited number of PDAC tissue samples. PDAC is a heterogeneous disease characterised by multiple genetic abnormalities, often present in many core signalling pathways [135], and so the differential expression seen in these limited samples may not be representative of the non-coding transcriptome. Although validation and investigation have been undertaken in 26 described candidates (see Table 1 and Table 6), this number is only a small proportion of the known circRNAs, and so investigation of other candidates may reveal additional clinical utility. With regards to CCA, a recent study undertaking high-throughput whole transcriptome sequencing in tissues hypothesised a further ceRNA network comprising of miR-144-3p, and 7 upregulated, and 10 downregulated circRNAs [136]. This miRNA has already been implicated in PDAC [86], and in this study, functional enrichment analyses suggest a downstream effect on the spliceosome, as well as on RNA processing and transport. This is one avenue that is opened for further work. Furthermore, the majority of circRNA work in PDAC has been undertaken by research groups based in China, with all tissue samples taken from Asian patients. It is not known to what degree circRNAs expression profiles differ between ethnic groups, and so these findings may not be generalisable to the Western world. “In Silico” and bioinformatical techniques have enabled contextualisation of circRNAs into predicted networks, however, it is important to be aware that hypothetical relationships require sound experimental interrogation.

Researchers have also considered the differential circRNA expression in other clinically useful samples other than tissue (i.e., blood and biofluids), however these avenues require further exploration. Blood sampling in particular may offer a simple, and non-invasive approach to investigating PDAC, potentially allowing real-time assessment of response to cancer treatment. However, only a limited number of circRNAs have been investigated in PDAC as blood-based biomarkers to date [47,79,82,116,127]. Other potentially useful biofluids for the discovery of circRNA biomarkers include bile, pancreatic cyst fluid and saliva. Differential circRNAs expression has been demonstrated in saliva for other malignancies [119]. In the context of pancreaticobiliary disease, bile and pancreatic cyst fluid can be obtained during standard clinical investigation at the time of endoscopic retrograde cholangio-pancreatography (ERCP) and endoscopic ultrasound (EUS), respectively. All current research has considered circRNAs expression as a diagnostic biomarker, however, these molecules may have further utility in prognosticating outcomes, predicting response to cancer treatments, and monitoring for disease progression or recurrence (e.g., after chemotherapy or surgical resection). Furthermore, it is important to recognise that PDAC is a heterogeneous disease process, and can develop from pre-malignant conditions (e.g., Intraductal Papillary Mucinous Neoplasms (IPMN) and chronic pancreatitis). Future work may consider the ability for circRNAs to differentiate between subtypes of PDAC, and predict the chance of malignant transformation of pre-malignant conditions.

The circRNA field is currently suffering from a lack of naming convention. In the early days of whole-transcriptome-based circRNA studies (from 2013 to 2015), circBase was the primary database supporting circRNA research [137], and as such circBase IDs were the preferred method for the identification of specific circRNAs. However, circBase has only seen minor updates after this initial period and is now no longer a comprehensive source of circRNA annotation. Other circRNA databases have subsequently arisen, such as CIRCpedia [138], and CircAtlas [139], which are now more up to date. However, each database implements its own circRNA ID. Even more worrying is the division of the circRNA field between sequencing-based studies, and Arraystar microarray-based studies (https://www.arraystar.com/), the latter implementing its own circRNA naming conventions. Arraystar does not offer the complete annotations for their circRNA probe set that would be needed to systematically compare sequencing data and microarray data, which effectively promotes a division of the circRNA field into microarray studies and sequencing studies.

Finally, the utility of circRNAs for liquid biopsy requires consideration in the context of other relevant biomarkers, including circulating tumour cells (CTCs), cell-free DNA, and other RNA molecules [140,141,142]. In addition, future studies should specifically consider the presence and utility of circRNAs in different compartments, including whole blood, plasma, serum, platelets, exosomes/EVs, and secretosomes.

## 4. Systematic Review Methodology

This systematic review was performed in keeping with the PRISMA guidelines. This review is based on previous study results and so no ethical approval or consent was required. This review was registered on the National Institute for Health Research (NIHR) PROSPERO database, identification number CRD42019156889.

### 4.1. Search

Medline, Embase, Scopus and PubMed were interrogated without limit on the time period with the following search strategy on the 16 April 2020. Both Title/Abstract and Medical Subject Heading (MeSH) were utilised (where available) for article identification, search strategy outlined below. Non-English and duplicated articles were excluded at this stage. Authors CL and AF performed title and abstract screening and for all identified articles, followed by full-text review. In addition, a manual search of identified reviews and referencing was performed to identify any additional relevant studies.

(Circular RNA OR circRNA) AND (Biliary tract cancer OR pancreatic ductal adenocarcinoma OR cholangiocarcinoma OR ampullary carcinoma OR Gallbladder carcinoma). For each term, common synonyms and MeSH headings were incorporated.

### 4.2. Eligibility Criteria

Articles types excluded at screening were those retracted, reviews, editorials, book chapters and conference abstracts. Additionally, those clearly not in the context biliary cancer or unrelated to the subject of interest were excluded.

On full text review inclusion required quantification of circRNA expression in the context of biliary tract cancer and evaluation against the feature of malignancy in vitro, in vivo or in the context of clinical outcomes.

### 4.3. Data Collection

Authors CL and AF performed data collection independently against a predefined database. After initial data collection, both authors undertook further data collection. For each article, the circRNA of interest was recorded along with related miRNA, mRNA, technique through which expression and relationships were validated; in vitro study outcomes; in vivo study outcomes; along with associated clinical tumour and patient evaluation.

### 4.4. Results Reporting

Meta-analysis was not performed. Data are presented in the subgroups of Pancreatic Ductal Adenocarcinoma and then other biliary tract malignancies.

## 5. Conclusions

Of all the non-coding RNA molecules, the abundance, stability and tissue specific expression patterns of circRNAs lend them to use in diagnostics, prognostics and molecular-targeted therapies for various diseases including PDAC. These features have only recently been realised, and so it is unsurprising that circRNAs have received increased attention over the past few years. The differential expression profile of circRNAs in malignancies such as PDAC has now been demonstrated, with validation of several molecules of interest. The majority of investigated circRNAs in PDAC are upregulated and are associated with malignant cellular processes such as proliferation, invasion and metastasis. The proposed oncogenic mechanism is predominantly through competitive inhibition of miRNAs, frequently termed “miRNA sponge”, through the ceRNA network. Other proposed mechanisms include interaction with RBPs, protein and peptide regulation, and pseudogene generation. It is ultimately likely to be through a range of actions dependant on the specific circRNA molecules in question, with further work required to expand this understanding. Bioinformatic analysis has enhanced our ability to understand the interaction of circRNAs within genetic and molecular pathways, but has also served to illustrate the complexity of these networks.

Like any investigation, the consideration and implementation of circRNAs as biomarkers for PDAC must proceed with caution. Despite the demonstrated significance in diagnostics and prognostics, there is still large room for error. The role of circRNAs must be considered in the wider context of each disease stage, and the treatments given. Ultimately, the future of PDAC diagnosis, prognosis and treatment is likely to be planning individualised care to each patient, and although research is starting to granulate the role of circRNAs and the ceRNA network in PDAC, it is clear that there is still a long way to go. Future research should continue to map out the differential circRNA expression profiles in PDAC, and establish how deregulated circRNAs are associated with tumour and clinical features. This should be expanded to include clinically useful samples such as blood, biopsies and biofluids. Evaluation of specific circRNAs that may have a role in diagnosis and prognostication should continue to be evaluated alone, and as a panel, as well as in combination with other diagnostic tests, including established tumour markers.

## Figures and Tables

**Table 1:** Summary of differentially expressed circRNAs evaluated in Pancreatic Ductal Adenocarcinoma (PDAC). * All studies evaluated PDAC patient samples except hsa_circ_100782 which was evaluated in PDAC cell lines alone.

Author	Year	Cancer Type	circRNA	Expression in PDAC Samples	Association with Features of Malignancy Demonstrated
Chen G. at al. [78] *	2017	PDAC	hsa_circ_100782	Upregulated	In vitro and in vivo
Yang F. et al. [79]	2017	PDAC	hsa_circ_0006988	Upregulated	Clinical data
An Y. et. al. [80]	2018	PDAC	hsa_circ_0099999 (circZMYM2)	Upregulated	In vitro and in vivo
Zhu P. et al. [81]	2018	PDAC	hsa_circ_0006215	Upregulated	In vitro
Li J. et al. [82]	2018	PDAC	circ-IARS	Upregulated	In vitro, in vivo and clinical data
Li Z. et al. [47]	2018	PDAC	circ-PDE8A	Upregulated	In vitro, in vivo and clinical data
Jiang Y. et al. [83]	2018	PDAC	hsa_circ_0001649	Downregulated	In vitro and clinical data
Qu S. et al. [46]	2019	PDAC	hsa_circ_0005397 (circ-RHOT1)	Upregulated	In vitro
Xu Y. et al. [84]	2019	PDAC	hsa_circ_0030235	Upregulated	In vitro and clinical data
Hao L. et al. [54]	2019	PDAC	hsa_circ_0007534	Upregulated	In vitro, in vivo and clinical data
Liu L. et al. [85]	2019	PDAC	ciRS-7 (Cdr1as)	Upregulated	In vitro and clinical data
Yang J. et al. [86]	2019	PDAC	hsa_circ_0007334	Upregulated	In vitro
Yao J. et al. [87]	2019	PDAC	circLDLRAD3	Upregulated	In vitro and in vivo
Chen Y. et al. [88]	2019	PDAC	circASH2L	Upregulated	In vitro, in vivo and clinical data
Xing C. et al. [89]	2019	PDAC	circADAM9	Upregulated	In vitro, in vivo and clinical data
Zhang X et al. [48]	2020	PDAC	hsa_circ_001653	Upregulated	In vitro, in vivo and clinical data
Liu Y. et al. [90]	2020	PDAC	circHIPK3	Upregulated	In vitro and in vivo
Wong C. et al. [91]	2020	PDAC	circFOXK2	Upregulated	In vitro and in vivo
Guo X. et al. [92]	2020	PDAC	hsa_circ_0009065 (circBFAR)	Upregulated	In vitro and in vivo
Kong Y. et al. [93]	2020	PDAC	hsa_circ_0086375 (circNFIB1)	Downregulated	In vitro, in vivo and clinical data
Guo W. et al. [94]	2020	PDAC	hsa_circ_0013912	Upregulated	In vitro, in vivo and clinical data
Zhang X. et al. [95]	2020	PDAC	hsa_circ_001587	Downregulated	In vitro, in vivo and clinical data

**Table 2:** Summary of investigated circRNAs in PDAC with proposed ceRNA network.

circRNA	Expression in PDAC Tissue	Expression in Cell Lines	miRNA	Implicated Molecules and Pathways
hsa_circ_100782	−	Upregulated	miR-124	IL6/STAT3
hsa_circ_0099999 (circZMYM2)	Upregulated	−	miR-335-5p	JMJD2C
hsa_circ_0006215	Upregulated	−	miR-378a-3p	SERPINA4
circ-IARS	Upregulated	Upregulated (and exosomes)	miR-122	ZO-1, RhoA, F-actin
circ-PDE8A	Upregulated	Upregulated	miR-338	MACC1/MET
hsa_circ_0005397 (circ-RHOT1)	Upregulated	Upregulated	miR-26b; miR-125a, miR-330; miR-382	−
hsa_circ_0030235	Upregulated	Upregulated	miR-1253; miR-1294	−
hsa_circ_0007534	Upregulated	Upregulated	miR-625; miR-892b	−
ciRS-7 (Cdr1as)	Upregulated	−	miR-7	EGF/STAT3
hsa_circ_0007334	Upregulated	−	miR-144-3p; miR-577	MMP7
circ-LDLRAD3	Upregulated	Upregulated	miR-137-3p	Pleiotrophin
circASH2L	Upregulated	Upregulated	miR-34a	Notch 1
circADAM9	Upregulated	Upregulated	miR-217	PRSS3
hsa_circ_001653	Upregulated	Upregulated	miR-377	HOXC6
circHIPK3	Upregulated	Upregulated	miR-330-5p	RASSF1
circFOXK2	Upregulated	Upregulated	miR-942	YBX1 and hnRNPK; NUF2 and PDXK
hsa_circ_0009065 (circBFAR)	Upregulated	Upregulated	miR-34b-5p	MET
hsa_circ_0086375 (circNFIB1)	Downregulated	Downregulated	miR-486-5p	PIK3R1/VEGF-C
hsa_circ_0013912	Upregulated	Upregulated	miR-7-5p	−
hsa_circ_001587	Downregulated	Downregulated	miR-223	SLC4A4

**Table 3:** Summary table of cellular functions of circRNAs in PDAC. * All investigated circRNAs were found to be upregulated in PDAC except hsa_circ_0001649 and hsa_circ_0086375 (circNFIB1), which were found to be downregulated. “↑” indicates an increase; “↓” indicates a decrease; “←→” indicates no significant difference demonstrated; and “−“ indicates this measure was not reported.

circRNA	Study Type	Proliferation/Viability	Migration	Invasion	Apoptosis
hsa_circ_100782	Silencing of upregulated circRNA	↓	−	−	−
hsa_circ_0099999		↓	−	↓	↑
hsa_circ_0006215		←→	↓	−	−
circ-IARS		−	↓	−	−
hsa_circ_0005397 (circ-RHOT1)		↓	↓	↓	−
hsa_circ_0030235		↓	−	−	↑
hsa_circ_0007534		↓	↓	↓	↑
ciRS-7 (Cdr1as)		↓	−	↓	−
hsa_circ_0007334		−	−	−	−
circ-LDLRAD3		↓	↓	↓	−
hsa_circ_001653		↓	−	↓	↑
circHIPK3		↓	↓	↓	↑
circFOXK2		↓	↓	↓	↑
hsa_circ_0009065 (circBFAR)		↓	↓	↓	−
hsa_circ_0013912		↓	↓	↓	↑
hsa_circ_0099999	Overexpression of upregulated circRNA	↑	−	↑	↓
hsa_circ_0006215		↑	↑	−	↑
circ-IARS		−	↑	−	−
circ-PDE8A		−	↑	↑	−
hsa_circ_0030235		↑	↑	−	↓
hsa_circ_0007534		↑	↑	↑	↓
circASH2L		↑	↑	↑	−
circADAM9		↑	↑	↑	−
hsa_circ_001653		↑	−	↑	↓
circFOXK2		↑	↑	↑	−
hsa_circ_0009065 (circBFAR)		↑	↑	↑	−
hsa_circ_0086375 (circNFIB1)	Silencing of downregulated circRNA *	−	↑	−	−
hsa_circ_001587		↑	↑	↑	−
hsa_circ_0001649	Overexpression of downregulated circRNA *	↓	−	−	↑
hsa_circ_0086375 (circNFIB1)		−	↓	−	−
hsa_circ_001587		↓	↓	↓	−

**Table 4:** Function of circRNAs in PDAC identified in animal studies. * All circRNAs were upregulated in PDAC, except for hsa_circ_0086375 (circNFIB1) and hsa_circ_001587, which were downregulated.

circRNA	Animal	Method	Findings
hsa_circ_100782	Nude mice	circRNA knockdown	Decreased tumour size
hsa_circ_0099999	Nude mice	circRNA knockdown	Decreased tumour size
circ-IARS	Nude mice	circRNA overexpression	Increased tumour size and metastatic disease
circ-PDE8A	Nude mice	circRNA overexpression	Increased peripheral blood exosomal GFP signals
hsa_circ_0007534	Nude mice	circRNA knockdown	Decreased tumour size
circ-LDLRAD3	Nude mice	circRNA knockdown	Decreased tumour size and weight
circASH2L	Nude mice	circRNA overexpression	Increased tumour size and metastatic disease
circADAM9	Nude mice	circRNA knockdown	Decreased tumour size and weight
hsa_circ_001653	Nude mice	circRNA knockdown	Decreased tumour size and weight
circHIPK3	Nude mice	circRNA knockdown	Decreased tumour size and weight
circFOXK2	Nude mice	circRNA knockdown	Decreased tumour size and metastasis
hsa_circ_0009065 (circBFAR)	Nude mice	circRNA overexpression	Increased tumour size
hsa_circ_0086375 (circNFIB1)	Nude mice	circRNA knockdown *	Increased lymph node metastasis
hsa_circ_0013912	Nude mice	circRNA knockdown	Decreased tumour size and weight
hsa_circ_001587	Nude mice	circRNA overexpression *	Decreased tumour size and weight
		circRNA knockdown *	Increased tumour size and weight

**Table 5:** Summary table of clinical characteristics seen with differential circRNA expression in PDAC. “↑” indicates an increase; “↓” indicates a decrease; “←→” indicates no significant difference demonstrated; and “−“ indicates this measure was not reported.

circRNA	Direction of Dysregulation	Sample Assessed	Tumour Size	Duodenal Invasion	Neural Invasion	Lymphatic Spread	Vascular Spread	Metastatic Disease	Stage (TNM)	Differentiation Grade	Survival Time
hsa_circ_0006988	Upregulated	Tissue	←→	−	−	↑	↑	←→	←→	−	−
hsa_circ_0006988	Upregulated	Plasma	←→	−	−	↑	↑	←→	−	−	−
circ-IARS	Upregulated	Tissue	←→	←→	↑	←→	↑	↑	↑	−	↓
circ-PDE8A	Upregulated	Tissue	←→	←→	←→	↑	←→	←→	↑	←→	↓
circ-PDE8A	Upregulated	Plasma exosome	←→	↑	←→	←→	↑	↑	↑	←→	↓
hsa_circ_0001649	Downregulated	Tissue	−	−	−	←→	−	−	↑	↓	↓
hsa_circ_0030235	Upregulated	Tissue	−	−	−	↑	−	−	↑	←→	↓
hsa_circ_0007534	Upregulated	Tissue	−	−	−	↑	−	−	↑	←→	−
ciRS-7 (Cdr1as)	Upregulated	Tissue	←→	−	−	↑	↑	−	−	−	−
circASH2L	Upregulated	Tissue	←→	←→	←→	↑	←→	←→	↑	←→	↓
circADAM9	Upregulated	Tissue	−	−	−	↑	−	−	↑	−	↓
hsa_circ_001653	Upregulated	Tissue	−	−	−	−	−	−	−	−	↓
hsa_circ_0009065 (circBFAR)	Upregulated	Tissue	←→	−	−	←→	−	−	↑	←→	↓
hsa_circ_0086375 (circNFIB1)	Downregulated	Tissue	←→	−	−	↑	−	−	↑	←→	↓
hsa_circ_0013912	Upregulated	Tissue	←→	−	−	↑	−	−	↑	−	−
hsa_circ_0086375 (circNFIB1)	Downregulated	Tissue	−	−	−	↑	−	−	−	↑	↓

**Table 6:** Summary of differentially expressed circRNAs investigated in biliary tract cancers.

Author	Year	Cancer Type	circRNA	Expression in Tumour Tissue	Ass Association with Features of Malignancy Evaluated
Xu Y et al. [98]	2018	CCA	hsa_circ_0001649	Down	In vitro, in vivo and clinical data
Jiang X et al. [96]	2018	CCA	ciRS-7 (Cdr1as)	Up	Clinical data
Kai D et al. [26]	2018	GBC	hsa_circ_0000284 (or circHIPK3)	Up	In vitro
Xu Y et al. [97]	2019	CCA	circ_0005230	Up	In vitro, in vivo and clinical data
Wang et al. [53]	2019	GBC	hsa_circ_0008234 (or circFOXP1)	Up	In vitro, in vivo and clinical data
Lu Q and Fang T [103]	2019	CCA	circSMARCA5	Down	In vitro and clinical data
Huang X et al. [33]	2019	GBC	circERBB2	Up	In vitro, in vivo and clinical data
Wang S et al. [100]	2019	CCA	hsa_circ_0000284	Up	In vitro and in vivo
Li D et al. [99]	2020	CCA	ciRS-7 (Cdr1as)	Up	In vitro and in vivo
Xu Y et al. [101]	2020	CCA	hsa_circ_102064 (or circ-CCAC1)	Up	In vitro, in vivo and clinical data
